# Clinical evaluation of a multiplex PCR-based test for joint infection: a prospective diagnostic accuracy study of forty-nine patients

**DOI:** 10.1007/s00590-024-04114-2

**Published:** 2024-10-02

**Authors:** Jacob Lund-Andersen, Matilde L. H. Petersen, Krassimir Kostadinov, Lennart Friis-Hansen, Henrik Calum, Søren Overgaard

**Affiliations:** 1 Department of Orthopedic Surgery and Traumatology, Copenhagen University Hospital, Bispebjerg and Frederiksberg, Copenhagen, Denmark; 2 Department of Clinical Biochemistry, Copenhagen University Hospital, Bispebjerg and Frederiksberg, Copenhagen, Denmark; 3 Center for Translational Research, Bispebjerg and Frederiksberg Hospital, Copenhagen, Denmark; 4 Department of Clinical Research, University of Southern Denmark, Odense, Denmark; 5 Department of Clinical Microbiology, Copenhagen University Hospital, Rigshospitalet, Copenhagen, Denmark; 6 Department of Clinical Microbiology, Copenhagen University Hospital, Amager and Hvidovre, Copenhagen, Denmark; 7 Department of Clinical Medicine, Faculty of Health and Medical Sciences, University of Copenhagen, Copenhagen, Denmark

**Keywords:** Joint infection, Diagnostic accuracy, PCR, Culture growth, Pathogen detection

## Abstract

**Purpose:**

The purpose of this study was to evaluate the diagnostic accuracy (sensitivity, specificity, positive predictive value (PPV) and negative predictive value (NPV)) of the PCR-based BioFire® Joint Infection Panel (BJI Panel) against microbiological culture growth for patients suspected of having a native or prosthetic joint infection.

**Methods:**

Synovial fluid and tissue biopsies were prospectively collected from patients from June 2022 to June 2023. The results of the BJI Panel were compared with those of culture growth.

**Results:**

51 samples were included. Including all pathogens, the sensitivity was 69%, the specificity 89%, the PPV 73% and the NPV 86%. Including only pathogens in the BJI Panel, the sensitivity was 100%, the specificity 90%, the PPV 73% and the NPV 100%.

**Conclusion:**

The BJI Panel has a high accuracy for detecting the pathogens in its panel, but the absence of important common pathogens from the panel reduces its sensitivity and NPV. With a short turnaround time and precise pathogen detection, the BJI Panel has the potential to add value as a complementary diagnostic method.

**Supplementary Information:**

The online version contains supplementary material available at 10.1007/s00590-024-04114-2.

## Introduction

Diagnosing the pathogen in a native or prosthetic joint infection (PJI) is often challenging [1]. The crucial part is microbiological culture growth of synovial fluid or tissue biopsies but other criteria are also included in the diagnose [2–5]. Culture of samples has limitations, including a process time of several days or weeks and susceptibility to antibiotics [5–7]. Moreover, its sensitivity and specificity vary with sensitivities ranging from around 50 to 90% and specificities ranging from around 75–100% [8–11].

New diagnostic methods have emerged like polymerase chain reaction (PCR) technology. It has the potential to mitigate these limitations through a short turnaround time and high sensitivity [8, 11]. Recently, a multiplex nucleic acid-based PCR diagnostic test panel (BioFire Joint Infection Panel) (hereafter BJI Panel) has been introduced on the market.

The aim of this study was to evaluate the diagnostic accuracy (sensitivity, specificity and predictive values) of the BJI Panel against our standard procedure of culture growth in a clinical setting for patients suspected of having a native joint infection or PJI.

## Methods

### Study design

The study was performed as a prospective cohort study at the Copenhagen University Hospital, Bispebjerg and Frederiksberg, Department of Orthopedic Surgery and Traumatology from June 2022 to June 2023. Patients with a suspected native joint infection or PJI and who underwent synovial fluid aspiration for culture growth as part of standard clinical care were eligible for inclusion.

The study was approved as a quality evaluation project at Copenhagen University Hospital, Bispebjerg and Frederiksberg, and Copenhagen University Hospital, Amager and Hvidovre (project no. 22067667/10751605).

The study is reported according to the 2015 STARD guidelines [12].

### Test methods

#### BJI Panel

The BJI Panel (BioFire®, bioMériux, Salt Lake City, USA) is a random-access point-of-care multiplexed nucleic acid amplification-based in vitro diagnostic test that combines nested multiplex PCR and high-resolution melting analysis to detect and identify nucleic acid targets from pathogens. The BJI Panel has been FDA cleared and has a CE mark under IVDR.

All tests were performed according to the manufacturer’s instructions using the BioFire 2.0 instrument at the Department of Clinical Biochemistry (Copenhagen University Hospital, Bispebjerg and Frederiksberg).

A minimum of 0.2 mL of synovial fluid aspirated under sterile conditions for culture growth as part of standard clinical care either before surgery was decided (preoperatively) or during surgery (perioperatively) was used for the BJI Panel, thereby avoiding any additional invasive procedures for the patient.

The BJI Panel tests for the presence of 31 pathogens (15 g positive, 14 g negative bacteria and two yeasts strains) and eight antimicrobial resistance genes (see supplementary Tables 1 and 2 in Online Resource 1) with a turnaround time of approximately one hour [13]. The BJI Panel was considered positive if any of the 31 pathogens were identified. Furthermore, it was noted if any of the eight antimicrobial resistance genes were identified.

#### Sample collection

For pre- and perioperative sampling, synovial fluid from the same joint aspirate used for the BJI Panel was used for culture growth. For perioperative sampling, five perioperative tissue samples obtained from the same procedure as the synovial fluid were additionally used for culture growth (four patients only had perioperative tissue biopsies and no synovial fluid culture, as a reference).

#### Microbiological culture growth

Samples for culture growth were sent to the Copenhagen University Hospital, Amager and Hvidovre, Department of Clinical Microbiology, which is responsible for microbiological testing for the Department of Orthopedic Surgery and Traumatology at Copenhagen University Hospital, Bispebjerg and Frederiksberg, and culture growth was conducted in accordance with local instructions. Synovial fluid samples were Gram-stained for microscopy and plated onto 5% blood agar (Statens Serum Institut, Copenhagen, Denmark (SSI)), chocolate plates (Department of Clinical Microbiology, Herlev Hospital, Copenhagen, Denmark (CMH)) and anaerobe plates (SSI). The 5% blood agar was incubated in CO_2_ at 35 °C for 2 days and examined every day. The anaerobe plates were incubated for 2 days in an anaerobic incubator and examined on day two. Furthermore, the synovial fluid samples were incubated in thioglycolate broth (SSI) and examined on day one, two and five.

The tissue samples were plated onto 5% blood agar, eosin-methylene blue agar (CMH) and anaerobe plates. The 5% blood agar and blue agar plates were examined on day one and two; the anaerobe plates were examined on day 2. Furthermore, the tissue samples were incubated in thioglycolate broth and examined on day one, two and five. On day five, the thioglycolate broth was plated onto an anaerobic plate if negative. The time of incubation was 7 days. In total, the tissue samples were incubated for 12 days. One of the tissue samples was also Gram-stained and examined under microscope. Matrix-assisted laser desorption ionization time of flight (MALDI-TOF, Brückner, Germany) was used for identification of bacteria. Müller–Hinton agar (CMH) was used for antibiotic susceptibility testing and was incubated in CO_2_ at 35 °C for 2 days.

For preoperative sampling, culture growth was considered positive if growth of the synovial fluid was positive. For perioperative sampling, culture growth was considered positive if either a minimum of two biopsies or one biopsy and the synovial fluid sample were positive for the same microorganism with an identical antibiotic susceptibility pattern. When reporting the results and listing the detected pathogens, culture growth was numerically considered as one sample irrespective of whether it consisted of synovial fluid, synovial fluid plus five tissue biopsies or only five tissue biopsies and if the same pathogen was detected in both the perioperative synovial fluid and in one or more biopsies, it was only counted as one pathogen.

### Analysis

#### Accuracy

Three calculations of the accuracy parameters sensitivity, specificity, positive predictive value (PPV) and negative predictive value (NPV) using the BJI Panel as index test and microbiological culture growth as reference were performed. The first included all pathogens and both synovial fluid and biopsy cultures as reference. The second included only pathogens in the BJI Panel (on-panel pathogens), accordingly we considered cultures positive for pathogens not in the BJI Panel (off-panel pathogens) to be negative for this calculation. The third included all pathogens but we only included culture of the synovial fluid as reference, i.e., all culture of biopsies were disregarded.

Furthermore, for the first calculation including all pathogens we did a separate sub-analysis for the two subgroups preoperative sampling and perioperative sampling.

#### Resistance detection

We counted and compared the number of samples with resistance detected by the BJI Panel and by culture growth. As the BJI Panel tests for eight specific antimicrobial resistance genes related to certain specified pathogens, while microbiological culture growth tests for resistance based on exposure of the pathogen to the antibiotic without specifying the genetic resistance mechanism, culture growth cannot meaningfully be used as reference for the BJI Panel. Thus, no calculations of accuracy were made for resistance detection.

For resistance detection, a sample was considered positive if a pathogen was detected in one of the five biopsies. Therefore, some samples were defined as negative for the BJI Panel accuracy calculations and as positive for the resistance calculations.

#### Statistics

Sensitivity, specificity, PPV and NPV were calculated using the formulas: Sensitivity = True positives/(true positives + false negatives). Specificity = True negatives/(true negatives + false positives). PPV = (sensitivity × prevalence)/((sensitivity × prevalence) + (1 − specificity) × (1 − prevalence)). NPV = ((specificity × (1 − prevalence))/((1 − sensitivity) × prevalence + specificity × (1 − prevalence)).

95% confidence intervals for sensitivity, specificity, PPV and NPV were calculated using Wilson’s method [14].

All calculations were performed in RStudio 2022.07.1.

## Results

### Patients

**Fig. 1:**
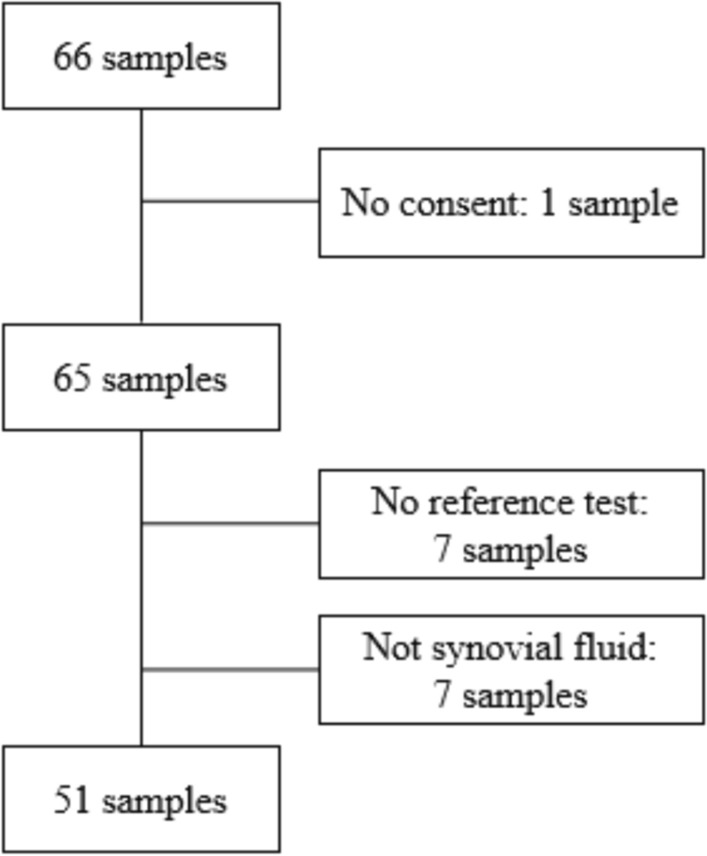
Patient cohort flowchart

66 individual samples of synovial fluid were obtained from 61 patients. (Five patients had the BJI Panel test done two times on separate occasions.) Of these, 51 synovial fluid samples from 49 patients were included in our study (Fig. 1).

The mean age of patients was 72 and 67 years for arthroplasty and native joint, respectively (Table 1). A majority of 44 samples were from prosthetic joints and 7 from native joints. 27 samples were from the hip, 23 from the knee and one from the foot. We obtained 26 samples preoperatively and 25 perioperatively.

**Table 1:** Patient characteristics

Characteristic	N	Overall, *N* = 51	Arthroplasty, *N* = 44	Native joint, *N* = 7
Age [yrs]	51	72 (11)	73 (9)	67 (22)
Joint	51			
Foot		1 (2.0%)	0 (0%)	1 (14%)
Hip		27 (53%)	24 (55%)	3 (43%)
Knee		23 (45%)	20 (45%)	3 (43%)
Reference test	51			
Perioperative biopsies × 5		4 (7.8%)	3 (6.8%)	1 (14%)
Perioperative synovial fluid and Perioperative biopsies × 5		21 (41%)	21 (48%)	0 (0%)
Preoperative joint aspiration synovial fluid		26 (51%)	20 (45%)	6 (86%)
Mean (SD); *n* (%)				

### BJI Panel and culture growth results

Across the 51 samples, the BJI Panel detected 19 pathogens and culture growth detected 24 pathogens (Table 2).

**Table 2:** Pathogens detected by BJI Panel and culture growth

Pathogen	Index test: BJI Panel (*N* = 51)	Reference test: Culture growth (SF and biopsies) (*N* = 51)	Reference test: culture growth(by sample type)		
			Preoperative SF (*N* = 26)	Perioperative SF (*N* = 21)	Perioperative biopsies × 5 (*N* = 25)
*In BJI Panel (on-panel pathogens)*					
Anaerococcus prevotii/vaginalis	1	0	0	0	0
Enterococcus faecalis	1	1	1	0	0
Finegoldia magna	1	1	1	0	0
Peptoniphilus	1	0	0	0	0
Staphylococcus aureus	6	6	2	2	4
Staphylococcus lugdunensis	1	1	0	1	1
Streptococcus spp.	6	0	0	0	0
Streptococcus agalactiae	2	1	0	0	1
Streptococcus pyogenes	0	1	0	0	1
Streptococcus dysgalactiae*	N/A	2	1	1	1
Streptococcus mitis*	N/A	1	1	0	0
Streptococcus sanguinis*	N/A	1	1	0	0
*Total on-panel pathogens*	19	15	7	4	8
*Not in BJI Panel (off-panel pathogens)*					
Corynobacterium species c. imitans	N/A	1	0	0	1
Staphylococcus caprae	N/A	1	0	0	1
Staphylococcus capitis	N/A	2	0	1	2
Propionibacterium acnes	N/A	1	0	0	1
Staphylococcus epidermidis	N/A	3	0	1	3
Staphylococcus hominis	N/A	1	0	0	1
*Total off-panel pathogens*	0	9	0	2	9
*Total on-panel* + *off-panel pathogens*	19	24	7	6	17

*N* number of samples, *SF* Synovial fluid, *N/A* Not Applicable

^*^Not specifically in the BJI Panel, but covered by the streptococcus spp. category in the panel

Staphylococcus aureus and streptococcus spp. were the most common pathogens detected by the BJI Panel. Among the off-panel pathogens detected by culture growth, none was detected in synovial fluid from preoperative sampling, two were detected in synovial fluid from perioperative sampling and nine were detected in perioperative biopsies. Staphylococcus epidermidis was the most prevalent off-panel pathogen detected by culture growth (see Online Resource 2 for full dataset).

### Accuracy-all pathogens

Including all pathogens, the sensitivity of the BJI Panel was 69% and the specificity was 89%. With a prevalence of infections in our sample population of 31%, the PPV was 74% and the NPV 86% (Table 3).

**Table 3:** Sensitivity, specificity, PPV and NPV of the BJI Panel including all pathogens with 95% confidence interval (CI) in brackets

BJI Panel	Reference: Culture Growth			
	Positive	Negative	Total	Accuracy
Positive	11	4	15	PPV: 73% (95% CI: 48–89)
Negative	5	31	36	NPV: 86% (95% CI: 71–94)
Total	16	35	51	
Accuracy	Sensitivity: 69% (95% CI: 44–86)	Specificity: 89% (95% CI: 74–95)		

Our subgroup analysis showed that for the preoperative sampling subgroup, the sensitivity was 100% (95% CI: 61–100), while it was only 50% (95% CI: 24–76) for the perioperative sampling subgroup, as all five positive culture samples not detected by the BJI Panel were in the perioperative subgroup. The NPV was 100% (95% CI: 82-100) for preoperative sampling and 74% (95% CI: 51–88) for perioperative sampling.

### Accuracy-on-panel pathogens

Including only on-panel pathogens, the sensitivity increased to 100% (95% CI: 74–100), as all pathogens detected by culture growth, but not by the BJI Panel, were off-panel pathogens. The specificity was 90% (95% CI: 77–96), the PPV was 73% (95% CI: 48–89) and the NPV increased to 100% (95% CI: 90–100).

### Accuracy-only synovial fluid

Including all pathogens, but using only synovial fluid as reference, 10 out of 12 positive culture samples were detected by the BJI Panel, giving a sensitivity of 83% (95% CI: 55–95). The two positive cultures not detected by the BJI Panel were off-panel pathogens. The specificity was 89% (95% CI: 74–95), the PPV was 71% (95% CI: 45–88) and the NPV was 94% (95% CI: 80–98).

### Resistance

The BJI Panel did not detect resistance genes in any of the samples (six out of the 15 positive BJI Panel results were positive with a pathogen which the BJI Panel tests for resistance genes, all six were staphylococcus aureus). Microbiological culture growth identified pathogens with antibiotic resistance in 13 out of the 19 positive culture samples.

## Discussion

The study aimed to evaluate the diagnostic accuracy of the BJI Panel in a clinical setting for patients suspected of having a native joint infection or PJI.

We showed that the BJI Panel had a sensitivity of 100% for on-panel pathogens; however, including all pathogens, the sensitivity was reduced to 69% because five out of 16 culture growth samples were positive for off-panel pathogens. All five were perioperative samples in which an off-panel pathogen was detected either in the biopsies taken during surgery (three samples) or in both the biopsies and synovial fluid (two samples). This reduces the sensitivity and the NPV (86%) of the test. Other studies of the BJI Panel have made similar observations, finding sensitivities in the range of 90–100% for on-panel pathogens but lower sensitivities, ranging from 30 to 83%, when including off-panel pathogens [2, 15–18]. Esteban et al. question the relevance of the off-panel pathogens [19]. In our study, one of the five patients with a culture positive with an off-panel pathogen was a clinically infected recently made knee arthroplasty positive for staphylococcus epidermidis.

Our study showed a higher sensitivity for the preoperative sampling subgroup than for the perioperative sampling subgroup (100% and 50%, respectively) and a higher sensitivity when using only culture of synovial fluid as reference than when also including culture of biopsies as reference (83% and 69%, respectively). These differences appear to be explained by off-panel pathogens being more common in samples obtained perioperatively as well as by more pathogens being detected by the growth of perioperative biopsies than by synovial fluid. This suggests that the BJI Panel potentially could have greater clinical benefit in the preoperative setting as complementary diagnostic to synovial fluid growth than in the perioperative setting. This is comparable to a different subgroup analysis by Schoenmakers et al. who found their results to indicate a clear clinical benefit of the BJI Panel for septic arthritis and late acute PJI (sensitivity of 83% and 73%, respectively), but a low clinical benefit for early acute PJI (sensitivity of 30%), due to the higher presence of infections with off-panel pathogens in this group [2].

The fast turnaround time of approximately 1 h for the BJI Panel compared to several days for culture growth has potential for optimizing antibiotic treatment and providing an advantage in patient treatment based on early pathogen and/or resistance detection. Hoffman et al. retrospectively found that the BJI Panel results would have had a substantial clinical impact on 51% of infected cases and 11% of non-infected cases [16], while Berinson et al. also retrospectively found a potential for optimization based on the rapid BJI Panel result [17]. Our study showed that the BJI Panel did not detect any of the resistance genes in the panel, while culture growth detected pathogens resistant to specific antibiotics in 13 out of 19 samples, and as such culture growth provided more information on pathogen resistance.

Our study has certain limitations. With 31 pathogens in the BJI Panel, only a small number of those pathogens were detected in our study and the sensitivity and specificity for other pathogens may be different. Second, we used culture growth as reference standard for positive infection, which has limited sensitivity and specificity. Accordingly, the sensitivity and specificity should be interpreted relative to microbiological culture growth. Furthermore, we have not made any distinction between patients with and without antibiotic use prior to sampling, and we did not use further PCR testing to follow up on results that were different for the BJI Panel and culture growth, which could otherwise have impacted the specificity that was lower than in comparable studies (we found a specificity of 89%, while other studies have reported specificities close to 100%) [2, 15–17, 19]. Finally, the BJI Panel does not include certain pathogens, which we found in culture growth in our study.

## Conclusion

The BJI Panel has a high accuracy for detecting the pathogens included in its panel, but the absence of important common pathogens from the panel, such as staphylococcus epidermidis, reduces the sensitivity and the NVP of the test.

The BJI Panel has the potential to add value as a complementary diagnostic method especially for preoperative synovial fluid culture growth, where the short turnaround time and precise pathogen detection can potentially lead to optimized antibiotic treatment.

## Supplementary Information

Below is the link to the electronic supplementary material.

Supplementary file1 (PDF 200 KB)

Supplementary file2 (XLSX 13 KB)

## Data Availability

The manuscript has data included as electronic supplementary material.
