# Supplementary material for: Clinical evaluation of a multiplex PCR-based test for joint infection: a prospective diagnostic accuracy study of forty-nine patients
Source: Eur J Orthop Surg Traumatol. 2024 Oct 2;34(8):4105–11. doi: 10.1007/s00590-024-04114-2 (PMC11519097; doi:10.1007/s00590-024-04114-2)
Supplement: Supplementary file 1 — Supplementary file1 (PDF 200 KB) [file 590_2024_4114_MOESM1_ESM.pdf]

Online Resource 1

Supplementary tables 1 and 2

Title: Clinical evaluation of a multiplex PCR based test for joint infection: A prospective diagnostic accuracy study of forty-nine patients

Journal name: European Journal of Orthopaedic Surgery & Traumatology

Authors: Jacob Lund-Andersen, Matilde LH Petersen, Krassimir Kostadinov, Lennart Friis-Hansen, Henrik Calum, Søren Overgaard

Corresponding author and affiliation: Jacob Lund-Andersen, jla_dk@hotmail.com, Copenhagen University Hospital, Bispebjerg and Frederiksberg, Department of Orthopedic Surgery and Traumatology, Copenhagen, Denmark

Supplementary table 1 BJI Panel targets and resistance testing [1]

GRAM-POSITIVE BACTERIA	GRAM-NEGATIVE BACTERIA	YEAST	ANTIMICROBIAL RESISTANCE GENES
Anaerococcus prevotii/vaginalis	Bacteroides fragilis	Candida spp.	**Carbapenemases**
Clostridium perfringens	Citrobacter	Candida albicans	IMP
Cutibacterium avidum/granulosum	Enterobacter cloacae complex		KPC
Enterococcus faecalis	Escherichia coli		NDM
Enterococcus faecium	Haemophilus influenzae		OXA-48-like
Finegoldia magna	Kingella kingae		VIM
Parvimonas micra	Klebsiella aerogenes		**ESBL**
Peptoniphilus	Klebsiella pneumoniae group		CTX-M
Peptostreptococcus anaerobius	Morganella morganii		**Methicillin Resistance**
Staphylococcus aureus	Neisseria gonorrhoeae		mecA/C and MREJ
Staphylococcus lugdunensis	Proteus spp.		**Vancomycin Resistance**
Streptococcus spp.	Pseudomonas aeruginosa		vanA/B
Streptococcus agalactiae	Salmonella spp.		
Streptococcus pneumoniae	Serratia marcescens		
Streptococcus pyogenes			

Supplementary table 2 Antimicrobial Resistance Genes and Applicable Bacteria [1]

BJI Panel Antimicrobial Resistance Gene Result	Enterococcus faecalis	Enterococcus faecium	Staphylococcus aureus	Staphylococcus lugdunensis	Citrobacter	Enterobacter cloacae complex	Escherichia coli	Klebsiella aerogenes	Klebsiella pneumoniae group	Morganella morganii	Proteus spp.	Pseudomonas aeruginosa	Salmonella spp.	Serratia marcescens
vanA/B	×	×												
mecA/C and MREJ (MRSA)			×											
CTX-M					×	×	×	×	×	×	×	×	×	×
IMP					×	×	×	×	×	×	×	×	×	×
KPC					×	×	×	×	×	×	×	×	×	×
NDM					×	×	×	×	×	×	×	×	×	×
OXA-48-like					×	×	×	×	×	×	×		×	×
VIM					×	×	×	×	×	×	×	×	×	×

References

1. BIoFire JI Panel Instructions for Use (RFIT-PRT-0690-01). https://www.biofiredx.qarad.eifu.online/ITI/US/en/all?keycode=ITI0017. Accessed 20-05-2024.
